# Successful treatment of dopamine dysregulation syndrome with dopamine D_2_ partial agonist antipsychotic drug

**DOI:** 10.1186/1744-859X-11-19

**Published:** 2012-07-07

**Authors:** Jin Mizushima, Keisuke Takahata, Noriko Kawashima, Motoichiro Kato

**Affiliations:** 1 Department of Neuropsychiatry, Keio University School of Medicine, 35 Shinanomachi, Shinjuku-ku, Tokyo, 160-8582, Japan; 2 Molecular Neuroimaging Program, Molecular Imaging Center, National Institute of Radiological Sciences, Chiba, Japan; 3 Kawashima Neurology Clinic, Kanagawa, Japan; 4 Department of Psychiatry, Komagino Hospital, Tokyo, Japan

**Keywords:** Dopamine dysregulation syndrome (DDS), Aripiprazole, Dopamine D_2_ partial agonistic antipsychotic drug, Parkinson's disease, Dopamine replacement therapy (DRT)

## Abstract

Dopamine dysregulation syndrome (DDS) consists of a series of complications such as compulsive use of dopaminergic medications, aggressive or hypomanic behaviors during excessive use, and withdrawal states characterized by dysphoria and anxiety, caused by long-term dopaminergic treatment in patients with Parkinson’s disease (PD). Although several ways to manage DDS have been suggested, there has been no established treatment that can manage DDS without deterioration of motor symptoms. In this article, we present a case of PD in whom the administration of the dopamine D_2_ partial agonistic antipsychotic drug aripiprazole improved DDS symptoms such as craving and compulsive behavior without worsening of motor symptoms. Considering the profile of this drug as a partial agonist at D_2_ receptors, it is possible that it exerts its therapeutic effect on DDS by modulating the dysfunctional dopamine system.

## Background

In the course of long-term antiparkinson therapy, patients with Parkinson’s disease (PD) often develop disinhibitory non-motor pathologies such as dopamine dysregulation syndrome (DDS) and impulse control disorders (ICD) including pathological gambling, hypersexuality and compulsive buying. Occasionally, punding or preoccupied repetitive behavior is also observed with DDS or ICD. DDS refers to excessive and compulsive use of dopaminergic medications well beyond the dose needed to control motor symptoms, and it has been typically recognized as a consequence of dopamine replacement therapy (DRT). Patients with DDS show aggressive or hypomanic behaviors during episodes of excessive use, as well as withdrawal states characterized by dysphoria and anxiety. DDS may improve with reduction of the dosage of the dopaminergic agent, but many patients are reluctant to decrease levodopa, showing an enduring tendency to relapse [1]. Here, we report the successful treatment of DDS in a PD patient with a dopamine D_2_ partial agonistic antipsychotic drug.

## Case presentation

The patient is a 63-year-old male who was diagnosed with PD at age 48, when he began suffering from shuffling gait of the right foot. He had no previous history of alcohol or drug abuse. He also had no history of smoking. Initially, he had been treated with amantadine and pramipexole. However, within six years, symptoms had worsened and rigidity of the whole body set in. Medication was then switched to levodopa/benserazide. Hoehn & Yahr Scale at this time was ranked as stage II. In the following years, he repeatedly complained of lower back pain and anxiety. Several medications including noritriptyline could not alleviate his pain, and only levodopa was beneficial. Nine years after beginning DRT, the patient started self-medication, using excessive doses of levodopa — up to 2000 mg a day. At the time he was referred to our hospital, he demonstrated compulsive use of levodopa and a withdrawal state including depressive symptoms such as anhedonia, loss of interest and back pain, with impaired social functioning. These depressive symptoms were not accompanied with psychotic symptoms. His family also reported punding, an intense preoccupation with repetitive tasks such as carpentry and gardening; he often started to strike nails with a hammer suddenly, continuing all day and into the night. EEG and brain MRI demonstrated no abnormal findings, and verbal and performance IQ on WAIS-R were 110 and 98, respectively. Based on the development of compulsive use through levodopa therapy, he was diagnosed with DDS.

At first, reduction of levodopa medication was initiated and the patient tried his best not to take excessive medications. However, he could not cease the excessive use of levodopa. At this point, treatment with aripiprazole at 6 mg/day was begun. By eleven months, with the dosage of aripiprazole having been raised to 18 mg/day, his craving for medications (daily dosages and intake intervals) had considerably subsided. After one year of aripiprazole therapy, the dose of levodopa was reduced to 600 mg/day without compulsive use, and no punding behavior was observed. His lower back pain also showed marked improvement, and his depressive symptoms diminished without worsening of motor symptoms. Adverse reactions related to aripiprazole including insomnia, akathisia, restlessness and sedation were not observed.

## Discussion

Within the past decade, DDS has become recognized as an important complication of dopamine replacement therapy, and several medical and surgical options for DDS have been suggested. One such option is subthalamic nucleus deep brain stimulation (DBS), which is accepted as an effective treatment of DDS [2]. However, DBS is not without risks, including side effects following surgery, impaired cognitive function and psychiatric complications [3]. Interventions including psychotherapy and social support are sometimes useful, but these have only a limited effect in the treatment of DDS. At present, the first line of treatment for DDS is generally medication rationalization. It has been reported that reduction in dopaminergic drug therapies could improve the disability caused by the behavioral disorders associated with DDS. However, dosage reduction of the dopaminergic agent can be troublesome due to worsening of motor symptoms and neuropsychiatric withdrawal symptoms. Furthermore, in the situation where patients may remain sensitized to the dopaminergic agent’s rewarding effects, it is often difficult to reduce its dosage. Therefore, a more effective and safe way to manage the addictive liability of the dopaminergic agent is needed. In this report, we described the therapeutic effect of a dopamine D_2_ partial agonistic antipsychotic drug on DDS in a patient with PD.

Several risk factors of DDS have been identified. The use of dopaminergic agents (especially chronic exposure to high doses of DRT), a previous history of mood disorders and alcohol dependence have shown to be relevant to the development of DDS in PD [4]. In our case, the patient had none of these risk factors except for high doses of levodopa, reportedly the most important risk factor for DDS. Although the exact mechanism underlying DDS remains unknown, it has been postulated that the addictive liability of dopaminergic agents is mediated by dysregulation of ventral striatal dopamine neurotransmission and abnormal activities of related neural circuitries that constitute the reward systems [5]. Supporting this hypothesis, human PET studies using ^11^ C]-raclopride showed enhanced drug-induced dopamine neurotransmission in the ventral striatum in PD patients with DDS compared to those without DDS [5]. Thus, alleviation of hyperdopaminergic neurotransmission in the ventral striatum is a potential target in the treatment of DDS.

In the present case, we used aripiprazole for the treatment of DDS. There were three reasons for this clinical choice. First, aripiprazole has a unique pharmacological profile that includes partial agonism at dopamine D_2_ receptors; it functions as an antagonist in conditions of high, but as an agonist in conditions of low activity of the dopamine D_2_ system [6].This means that aripiprazole may exert its therapeutic effect via modulation of the dopamine system by acting as antagonist during high dopaminergic tone and as agonist during the withdrawal state, and thus the dopamine D_2_ partial agonistic antipsychotic drug might represent an effective treatment choice for the compulsive use of levodopa.

The second reason for choosing aripiprazole is its very low risk of adverse effects on motor symptoms of PD [7]. It has been reported that even at full receptor occupancy by aripirazole, extrapyramidal side effects comparable to those expected with other antipsychotics such as haloperidol do not occur due to its intrinsic activity on dopamine D_2_ receptors [8]. Also, recent human PET studies of aripiprazole using ^11^ C]raclopride and ^11^ C]FLB457 indicate that its intrinsic dopaminergic signal due to its partial agonism at dopamine D_2_ receptors, rather than regional distribution of dopamine D_2_ receptor occupancy, contribute to the minimal risk of extrapyramidal side effects [9]. Thus, D_2_ partial agonistic antipsychotic drug displays its therapeutic effects with a minimum possibility of extrapyramidal side effects. On the other hand, aripiprazole should be used with caution for the treatment of psychosis in PD, because aripiprazole generally has the adverse effects of worsened motor signs and symptoms [10]. However, one previous study suggested that the low doses of aripiprazole might be tolerable in treatment of PD and display a significant decrease in the intensity and frequency of L-dopa-induced dyskinesias [11].

Thirdly, several studies have demonstrated the efficacy of the dopamine D_2_ partial agonistic antipsychotic drug against the compulsive use of alcohol and psychostimulants [12]. The core features of DDS are self-medication and levodopa-seeking behavior and hoarding associated with disabling mood, which share common characteristics of substance dependence. Given the putative action mechanisms of aripiprazole described above, as well as its target areas including the frontal-subcortical circuit that subserves reward/craving and compulsive behavior, it is hypothesized that the modulating effect of aripiprazole on the dysfunctional dopamine system contributed to the improvement of DDS symptoms. Thus, it may be considered that the use of a dopamine D_2_ partial agonistic antipsychotic drug presents a potentially therapeutic approach for DDS.

Several limitations to these preliminary results should be noted. First, our finding must be interpreted with some caution because it was based on a single case study. Second, the optimal dose setting of aripiprazole for the treatment of DDS has not been determined. Thus, clinical studies with a more systematic design and a greater number of patients are needed to elucidate the efficacy of aripiprazole for DDS.

## Conclusions

In our case, aripiprazole successfully improved DDS without worsening motor symptoms. To our knowledge, this is the first case report to present the efficacy of a dopamine D_2_ partial agonistic antipsychotic drug for DDS symptoms, and further clinical studies are encouraged.

### Consent

Written informed consent was obtained from the patient for publication of this case report. A copy of the written consent is available for review by the Editor-in-Chief of this journal. This study was done as part of standard care.

## Competing interests

The authors declare that they have no competing interests.

## Authors’ contributions

All authors contributed equally during the clinical evaluation and follow-up period. JM wrote the first draft of the manuscript, and all authors contributed to and have approved the final manuscript.
